# Migraine without Aura and Subclinical Atherosclerosis in Young Females: Is Gut Microbiota to Blame?

**DOI:** 10.3390/medicina55120786

**Published:** 2019-12-16

**Authors:** Doina Georgescu, Mircea Stefan Iurciuc, Ioana Ionita, Simona Dragan, Mihaela Muntean, Oana Elena Ancusa, Daniela Reisz, Mihai Ionita, Daniel Lighezan

**Affiliations:** 1 Department of Internal Medicine,“Victor Babes” University of Medicine and Pharmacy, 300041 Timisoara, Romania; mdioanaionita@yahoo.com (I.I.); michaelamuntean@yahoo.com (M.M.); oancusa@gmail.com (O.E.A.); ionitamihai79@yahoo.com (M.I.);; 2 Department of Cardiology,“Victor Babes” University of Medicine and Pharmacy, 300041 Timisoara, Romania; simona.dragan@umft.ro; 3 Department of Neurology,“Victor Babes” University of Medicine and Pharmacy, 300041 Timisoara, Romania; reisz_daniela@yahoo.com

**Keywords:** young female migraineurs, subclinical atherosclerosis, gut microbiota

## Abstract

*Background and Objectives:* Migraine with aura (MA) could be considered a risk factor for developing atherosclerosis and cardio-vascular events. However, less is known about the relation between migraine without aura (MWA) and atherosclerosis. Our study aimed to assess whether young female migraineurs, with alterations of gut microbiota could associate early atherosclerosis. *Materials and Methods:* We conducted an exploratory cross-sectional, pilot study concerning 105 consecutive young females having MWA, with recent normal brain scans, that were free of cardio-vascular risk factors, non-smokers, not on oral contraception, not pregnant, and without thyroid or parathyroid diseases, chronic organ failure, cancer, or on probiotic or antibiotic treatment. Consecutive to assessment of gut microbiota, patients were assigned to two groups: dysbiosis positive (*n* = 45) and dysbiosis negative (*n* = 60). All study participants underwent clinical examinations with an assessment of migraine severity, body mass index and carotid intima-media thickness (CIMT), as well as laboratory workups. Statistical analysis was performed using a chi-squared test (*χ*^2^), a two-tailed t-test and a nonparametric Spearman’s correlation test. *Results:* The dysbiosis positive migraineurs showed a significant increase in CIMT along with several anthropometrical, biological and clinical particularities. Significant positive correlations between dysbiosis and CIMT, glycosylated hemoglobin, migraine severity and duration, tumor necrosis factor-alpha, and body mass index were found. *Conclusions:* Young female migraineurs with significant alterations of gut microbiota experienced early signs of atherosclerosis and displayed severe migraine disability, as well as multiple biological and clinical particularities.

## 1. Introduction

Migraine with aura (MA) could be a cardio-vascular risk factor, even at younger ages. It seems that women complaining of MA are more likely to have associated traditional Framingham cardiovascular risk factors, increasing their risk for myocardial infarction, hypertension, dyslipidemia and obesity [1,2]. Recently, a prospective cohort study in young women having migraine, that were free of angina and cardio-vascular disease at enrolment revealed an important link between migraines and the risk of developing atherosclerosis (ATS) and cardio-vascular events, after more than 20 years of follow-up [3]. Although many studies have explored the association between MA and cardio-vascular risk factors, there is also evidence that migraine without aura (MWA) could represent a cardio-vascular risk, as well. A large case control study of the U.S. population, that assessed risk factors for cardio-vascular diseases (CVD) in individuals with MA and MWA and in controls, highlighted the highest risk in MA, but also elevated in MWA, with Framingham scores being significantly higher in MA and MWA than in controls [4]. Another large cross-sectional population-based study (HUNT) that examined the relationship between different kinds of headaches and cardiovascular risk factors came to the conclusion that both MA and MWA are associated with an unfavorable cardiovascular risk profile, probably with different underlying pathways [5].

Despite advances in medical knowledge and numerous studies performed over time, the etiology of ATS is still a subject of debate. ATS continues to be considered a multifactorial disease, with various risk factors, some genetic, others acquired, being involved in the emergence and progression of atherosclerotic plaque [6]. The relation between traditional risk factors and ATS has been considered a proportional equation, resulting in the stratification of cardio-vascular risk, but recent data reported a modest correlation between Framingham risk factors and severity of coronary artery disease, in adults with sudden coronary death [7]. Moreover, some coronary patients, especially younger adults and women, currently lack traditional Framingham cardio-vascular risk factors such as familial history, hypertension, dyslipidemia, diabetes mellitus, and smoking, highlighting the possibility of additional predisposing factors [8,9]. While inflammation is thought to be one of the most important steps in atherogenesis, several studies have demonstrated the importance of molecular components of various cells, e.g. the overexpression of monocyte chemoattractant protein 1(MCP-1) in endothelial cells, as well as in smooth muscle cells and macrophages, or the recruitment of different effectors with release of the proinflammatory cytokines IL-8 and TNF alpha [10,11]. Since the cornerstone of the atherosclerotic process is inflammation, various triggers to the immune response, that modulate the development and progression of the disease were also rigorously investigated. Some antigens capable to promote an immune response at the plaque level were analyzed, e.g. modified LDL and different kinds of proteins, including microbial antigens. The infectious hypothesis in ATS pathogenesis was explored, with different types of infections being linked to emerging of atherosclerotic plaque. Several pathogens, whether bacteria or viruses, were associated to ATS as a result of epidemiological studies and the identification of infectious agents in atherosclerotic tissues [12]. Recent research has focused on the imbalance of gut microbiota and its involvement in various aspects of pathology. Some dysfunctions of the intestinal ecosystem and subsequently gut-derived substances could play a role in the corruption of information, triggering inflammation and eventually resulting in various conditions, including hypertension (HTA) and ATS [13].

The relationship between gut dysbiosis and cardio-vascular risk was highlighted by several studies that linked gut microbiota imbalances to various CVDs. Some researchers concluded that gut flora metabolism could prevent CVD through the intervention of gut-derived metabolic substances [14]. Gut dysbiosis in relation to CVD was extensively analyzed. Some reviews clearly illustrated how gut dysbiosis is implicated in the development of ATS and hypertension, two major risk factors for CVD, via metabolite dependent and independent pathways [15,16]. A growing number of recent observations associated gut-derived metabolites such as methylamines, polyamines, short-chain fatty acids, trimethylamine N-oxide (TMAO) and secondary bile acids to obesity, type 2 diabetes, HTA and related metabolic diseases [17,18].

The primary aim of this study was to assess the association between gut microbiota dysbiosis and early ATS in young females with MWA, free of cardio-vascular risk factors.

The secondary aim of this study was to identify demographical, clinical and biological differences between dysbiosis-positive participants vs. dysbiosis-negative participants.

## 2. Materials and Methods

### 2.1. Methodology Addressing the Primary Aim

#### 2.1.1. Selection of Candidates for the Study

A group of 105 consecutive young inpatient females aged under, or equal to, 45 years joined this observational, cross-sectional, exploratory, pilot study, being recruited from a group of patients admitted for persistent headache, when the diagnostic of MWA was confirmed. The study took place at a university hospital, from 1 January 2017 to 1 January 2019, where 369 young female patients with the diagnostics of MWA were checked for eligibility. A group of 242 patients were excluded as they did not meet inclusion criteria. From 127 potential participants, 105 were confirmed eligible, who then undertook a microbiological stool exam, being assigned to two groups on the basis of gut dysbiosis (DB) presence: 45 DB positive, 60 DB negative.The inclusion criteria were a recent normal brain scan (in the last 6 months), and a normal ECG, chest X-ray, and echocardiography. All subjects had to be free of cardio-vascular risk factors: no familial history of myocardial infarction or stroke, no use of oral contraceptives, non-pregnant, nonsmoking, leading a non-sedentary lifestyle, no HTA (blood pressure-BP < 130/80 mmHg), no diabetes mellitus (fasting blood glucose < 126 mg%) or obesity (body mass index-BMI < 30 kg/m^2^), and no dyslipidemia (total cholesterol < 200 mg%, LDL < 100 mg%, HDL > 50 mg%, triglycerides < 150 mg%). Patients with thrombophilia, thyroid, parathyroid, or collagen diseases, chronic organ failure (pulmonary, cardiac, hepatic, renal), inflammatory bowel disease, celiac disease or gluten sensitivity, cancer, or undertaking ongoing treatment with antibiotics or probioticswere also excluded. All the study participants were subjected to the same protocol consisting of a thorough clinical examination assessing migraine severity, BMI and BP measurements. Laboratory workups were run using standard, accredited laboratory methods; measurements for CIMT were also performed. The study was approved by the Ethics Committeeof Scientific Research of the University of Medicine and Pharmacy “Victor Babes” Timisoara, Romania (Project identification code 18, approved on 28th of June 2019), and written informed consent was obtained from all participants before the beginning of the study.The study was conducted in accordance with the Declaration of Helsinki.

#### 2.1.2. Migraine Diagnostics

The criteria used for diagnosis of MWA (1.1), according to ICHD-2 were: (a) at least 5 attacks fulfilling criteria B–D; (b) headache attacks lasting 4–72 h and occurring ≥15 days/month (untreated or unsuccessfully treated); (c) headaches with at least 2 of the following characteristics—unilateral location, pulsating quality, moderate or severe pain intensity, and aggravated by or causing avoidance of routine physical activity (e.g., walking or climbing stairs); (d) during headache, there is at least 1 of the following—nausea and/or vomiting, photophobia and phonophobia; (e) not attributed to another disorder. For diagnosis of probable MWA (1.6.1), the criteria were: (a) attacks fulfilling all but 1 criteria from A–D for migraine without aura (1.1); (b) not attributed to another disorder [19]. All cases assigned for this study were examined by a trained neurologist and the diagnosis of MWA (1.1) was confirmed in all of the research participants. A thorough differential diagnosis of migraine was performed, other possible conditions being ruled out, one by one. Patients with probable MWA were not included in this study.

#### 2.1.3. Assessment of Migraine Attack Severity

Severity of migraine was established according to the Migraine Disability Assessment (MIDAS) questionnaire [20]. The MIDAS questionnaire was used to assess the disability related to headache during daily activities (work, home and family commitments, leisure or social activities). Questionnaires were completed under the supervision of trained nurses. Migraine disability was graded in four classes according to MIDAS scores: 0–5 minimal, 6–10 mild, 11–20 moderate, and ≥21 severe.

#### 2.1.4. Assessment of Gut Microbiota and Confirmation of Dysbiosis

Stool samples were collected in special sterile containers and sent to the laboratory in a maximum 2 h. The processing of stool samples aimed to determine whether species were aerobe, anaerobe or microaerophils, and to assess the colony forming units (CFU)/gram stool [21]. Species were identified by matrix-assisted laser desorption ionization using the MALDI-TOF-MS method [22]. Stool microbiological charts displayed either an increase or reduction in diverse microorganisms (+ modestly, ++ moderately and +++ greatly), as well as borderline situations (±), or physiological condition (normobiosis). Depending on the severity of gut microbiota’s imbalance charts, dysbiosis was scored as follows: mild (+) 1 point, moderate (++) 2 points and severe (+++) 3 points.

#### 2.1.5. Imaging Subclinical Atherosclerosis by Carotid Intima-Media Thickness

Extracranial Duplex examination was performed with a linear 10–12 MHz probe, on a high-resolution ultrasound equipment, in pain-free intervals, in order to assess the vascular damage, defined as the presence of carotid intima-media thickness (CIMT). CIMT > 0.9 mm is a marker of subclinical ATS according to The European Society of Cardiology (ESC) and European Society of Hypertension (ESH) 2013 guidelines SCORE Chart [23]. Patients were lying in a supine position, with the head rotated opposite to the examined region. After the visualization of the common carotid artery (CCA) from three angles (transversal, longitudinal anterior and posterior to thesterno-cleido-mastoid muscle), the CIMT was assessed 1 cm before bifurcation, at the posterior (far) wall, on both sides [24]. Three automatic measurements of CIMT for each angle were made and maximal values were recorded. The CIMT value considered for statistical analysis was represented by the mean of these primary measurements. In order to prevent significant inter-observer differences, a uniform protocol technique was applied, and ultrasound exams were performed by the same certified technician on the same equipment.

### 2.2. Methodology Addressing the Secondary Aim

#### 2.2.1. Assessment of Body Mass Index (BMI)

BMI was calculated based on patients height and weight, using the formula: BMI = weight (kg) height (m)^2^ and interpreted as underweight (≤18.5 kg/m^2^), normal (18.5–24.9 kg/m^2^), overweight (25.0–29.9 kg/m^2^), obese (30.0–39.9 kg/m^2^) or morbidly obese (≥40 kg/m^2^).

#### 2.2.2. Blood Pressure (BP) Measurements

BP measurements were performed in pain-free periods. At least 2 measurements of BP were taken in the morning, with patients at rest, in a sitting position, using the same standardized device. The final value of BP represented the mean of these primary two measurements.

#### 2.2.3. Blood, Urine and Stool Work Ups

Venous blood samples were collected early in the morning in fasting state and pain-free periods. Hemoglobin, white blood cells (WBC) and platelets (PLT) counts, biochemical exams including high sensitive C reactive protein (CRP), tumor necrosis factor-alpha (TNF-alpha), glycosylated hemoglobin (HbA_1_C), alanine-aminotransaminase(ALT), creatinine, calcemia, urine biochemistry and stool exams (calprotectin, lactoferrin, cytology and microbiology) were run using standard methods. An estimated glomerular filtration rate (e GRF) was calculated based on the Cockcroft–Gault formula, according to ADA (American Diabetes Association).

### 2.3. Statistical Analysis

Statistical analysis was performed using Graph Pad Prism 8.2 software (Graph Pad Software, Inc., La Jolla, CA, USA). Given an exploratory, pilot study, no previous, formal sample size calculations were performed. A chi-squared test (*χ*^2^) was used to compare the analyzed categorical variables between the two groups. A panel for continuous data was run for descriptive statistics and all mathematical numeric values were expressed as mean values (MV) and standard deviation (SD). A two-tailed t-test was performed in order to calculate p values with a confidence interval CI = 95%; *p* ≤ 0.05 was considered statistically significant. Nonparametric Spearman’s correlation test was also performed with the calculation of r coefficient and equation of linear regression between variables. The results were consecutively represented in graph format.

## 3. Results

This observational, cross-sectional, exploratory, pilot study was performed on 105 consecutive young inpatient females, diagnosed with MWA, divided into two groups: DB− and DB+. No statistically significant differences were recorded between the two groups concerning age, urban/rural location and marital situation. Statistically significant differences were observed in favor of the DB+ group related to higher levels of education, higher systolic arterial BP, but still within normal range, migraine duration and severity, as well as BMI. Data concerning these baseline demographic and clinical characteristics of patients are summarized in Table 1.

The blood count results, biochemistry parameters and Duplex measurements of CIMT are comparatively analyzed in Table 2. Statistically significant elevations of leukocytes,/ALT, ionic calcium, HbA_1_c and TNF-alpha were recorded in the DB+ group. However, leukocytes,/ALT and HbA_1_c elevations observed in DB+ group still remained within the normal ranges. Duplex measurements revealed a statistically significant increase in CIMT in the DB+ group, compared to in the DB−group (*p* < 0.0001), which exceeded the cutoff value of 0.9 mm in 40% of cases (*p* = 0.039).

**Figure 1:**
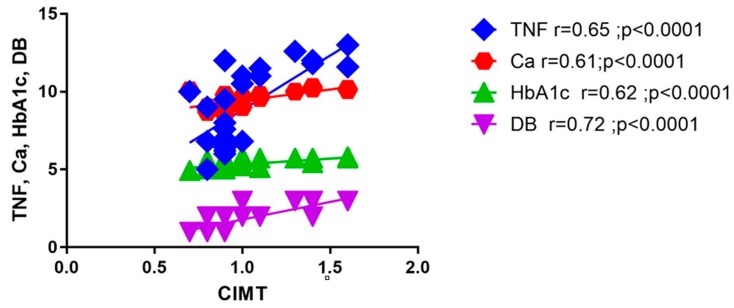
CIMT correlations with biological parameters. Ca = calcium, CIMT = carotid intima-media thickness, DB = dysbiosis, HbA_1_c = glycosylated hemoglobin, TNF-alpha = tumor necrosis factor-alpha.

The nonparametric Spearman’s correlation test revealed a close link of DB and TNF-alpha to CIMT, suggested by the strong positive correlations of CIMT to DB ranges (r = 0.72, *p* < 0.0001) and TNF-alpha levels (r = 0.65, *p* < 0.0001). Strong positive correlations were also found between CIMT, HbA_1_c and ionic calcium(r = 0.62, *p* < 0.0001 and r = 0.61, *p* < 0.0001). All these data are depicted in Figure 1.

**Figure 2:**
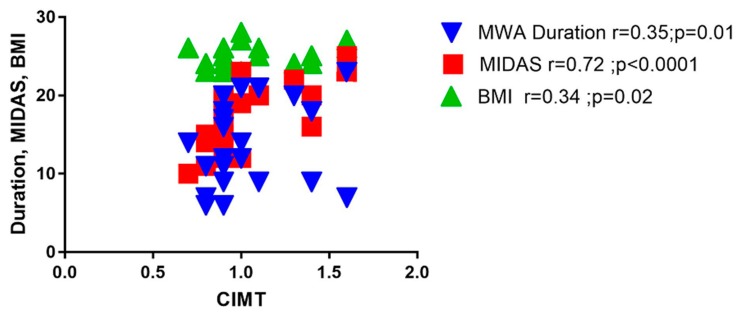
CIMT correlations with clinical parameters. BMI = body mass index, CIMT = carotid intima-media thickness, MWA-duration = migraine without aura-duration, MIDAS = migraine disability assessment score.

A strong positive correlation was observed between CIMT and MIDAS (r = 0.72, *p <* 0.0001), as well as good positive correlations between CIMT and MWA duration (r = 0.35, *p* = 0.01) and BMI (r = 0.34, *p =* 0.02), as depicted in Figure 2.

**Figure 3:**
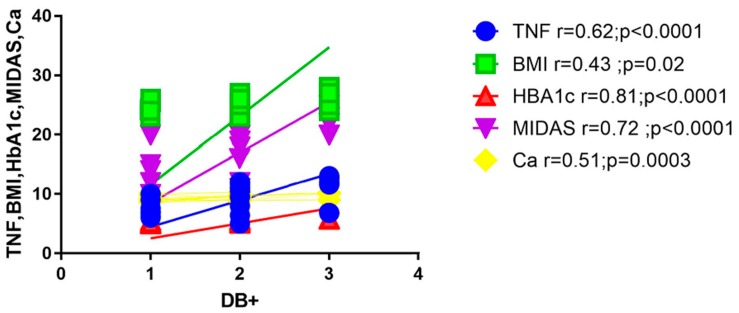
Dysbiosis correlations: BMI = body mass index, Ca = calcium, HbA_1_c = glycosylated hemoglobin. MIDAS = migraine disability assessment score, TNF-alpha = tumor necrosis factor-alpha.

In the DB+ group, strong positive correlations with HbA_1_c (r = 0.81, *p* < 0.0001) and, respectively, MIDAS (r = 0.72, *p* < 0.0001) were present. Statistically significant positive correlations were also noted with TNF alpha: r = 0.62, *p* < 0.0001, calcemia: r = 0.51, *p* = 0.0003 and BMI: r = 0.43, *p* = 0.02, as illustrated in Figure 3. These correlations were analyzed according to the severity of DB.

The DB+ group displayed different ranges of microbiota alteration as follows: 40% mild, 40% moderate and 20% severe.

All the significant correlations between the main variables, CIMT and DB, according to Spearman’s nonparametric test, were separately analyzed and are illustrated in table below (Table 3).

## 4. Discussion

It is thought that MA could influence cardio-vascular risk, even at younger ages, but MWA in young women free of traditional cardio-vascular risk factors represents a completely unexplored field for possible implications in subclinical ATS.

However, there is a lot of heterogeneity when it comes to the association of migraine with cardio-vascular risk, either related to major cardio-vascular events or to subclinical ATS. Many studies that evaluated ATS in migraineurs provided conflicting results, possibly as a consequence of not using the same protocol. Some studies evaluated all kind of headaches, not only migraines, and used different headache classifications; others recruited participants based only on self-reporting or questionnaire completion, without any clear confirmation of migraine diagnosis. Thus, a prospective study found no association of migraine to subclinical ATS [25]. Another case-control study suggested that the enhancement of CIMT could be associated with vasoactive antimigraine agents [26]. However, other researchers suggested, based on CIMT or epicardial fat and biological marker changes, that migraine (episodic and chronic) is associated with endothelial dysfunction and more severe migraine could be associated with important systemic endothelial damage [27,28,29].

The present study seems to be the first one to report that in relatively young (aged under 45 years) female patients with MWA, that are free of any known cardio-vascular risk factors, a significant increase in CIMT, as a marker of subclinical ATS, could be linked to gut DB. Similar observations were reported in the general population, by previous studies that emphasize the link between gut DB and cardio-vascular diseases [30].

Modified gut permeability, secondary to altered microbiota, could trigger inflammation involved in the onset of many conditions that consequently increase cardio-vascular risk. Our study revealed important correlations between TNF-alpha, CIMT and DB. The association between increases of inflammation markers and cardio-vascular risk has been highlighted before [31,32]. In accordance with recent research that emphasizes a link between microbiota particularities and overweight, obesity and metabolic syndrome—already known as risk factors for ATS—we also observed a positive correlation between DB severity and BMI in the studied female migraineurs population.

These microbiota modifications could be genetically induced or could be a result of local bacterial metabolism change, either as a result of nutrition or medication [33]. A couple of studies demonstrated a relationship between early exposure to antibiotics, the disruption of gut ecology and obesity in infant populations [34,35]. Some evidence also exists that BMI is a major covariate of microbiome variations and that obesity is associated with changes in intestinal microbiota composition [36]. Our study found a significant positive correlation between serum calcium levels, CIMT and DB severity, suggesting the possible involvement of gut DB in the management of intestinal calcium.

Many researchers reported a link between elevated calcium levels and cardio-vascular risk. Some authors reported an inverse paradoxical relationship between risk of ATS and bone mineral density [37]. The buildup of calcium components in the initially soft lipid ATS plaque is realized as a process of ectopic calcification by the local promotion of bone-like mineralization phenomena [38].

Recently, it has been hypothesized that gut DB could promote ATS via two main pathways: either metabolism-dependent or metabolism-independent. Endotoxemia and the increased presence of circulating lipopolysaccharides (LPS) could interfere to reverse cholesterol transport at the level of macrophages as a metabolism-independent pathway. DB could promote ATS by metabolism-dependent pathways related to the interference of bile acids metabolism and the generation of TMAO (trimethylamine-n-oxide) [39]. There are studies that even extrapolate the prediction for peripheral arterial disease based on some gut-derived metabolites, such as TMAO, which are seen as significant prognostic markers related to ATS in patients with coronary artery disease [40]. DB gut signatures linked to metabolic syndrome seem to be associated to a particular phenotype microbial imbalance, that results in an increase in circulating LPSs with emerging pro-inflammatory responses, decreasing insulin signaling, and that favors adiposity and obesity. A configuration of gut microbiota characterized by an increase in *Probacteria* and *E. coli* with a decrease in the *Firmicutes* population was reported in patients with nonalcoholic fatty liver disease and more severe stages of fibrosis [41]. It is possible that dysbiotic migraineur patients—primarily investigated in our study for ATS risk, who displayed concurrent slightly elevated levels of transaminases (ALT), HbA_1_c, and BMI in the overweight range, should already be associated with fatty liver disease.

It is equally possible for a particular gut microbiota imbalance to result in many coincidental consequences, including headaches, by intercepting the brain–gut axis, as we also reported in a previous study [42]. Since gut DB is related to obesity, insulin resistance, metabolic syndrome, HTA and ATS, many studies advocate the utility of prebiotics, probiotics and postbiotics, as new alternatives for approaching these conditions and subsequently managing cardio-vascular risk [43]. As a consequence, gut microbiota-targeted therapy could be taken into account for the prevention and treatment of CVD and migraine [44,45,46,47].

Limitations: This observational pilot study addresses the potential exposure to gut microbiota dysbiosis, in a particular group of patients—young females with MWA, providing preliminary data regarding a possible association of dysbiosis with early ATS. Given the cross-sectional study, a lot of statistical drawbacks could arise. Sources of potential information bias, referring to inaccurate assessment of exposure and related to measurements of DB magnitude, respectively, were as low as possible, whereas a uniform protocol of sample collection and transportation was applied and microbiological examination with DB chart report was performed in the same laboratory with the same methods. Considering outcome assessment as another potential source of information bias, measurements of CIMT were performed on the same equipment with the same operator, pursuing the same protocol. Information biases related to MWA diagnoses were very limited, since all participants were examined by a neurologist and the diagnosis was confirmed. MIDAS questionnaires and the way study participants correctly/incorrectly appreciated severity of disability, including recall bias could have interfered with the precision of the related data analysis and interpretation, but the completion of these questionnaires was made under the supervision of trained nurses. Other sources for potential bias and possible confounders were partly overcome by the study design, e.g., homogenous study participants (all women of the same age, MWA diagnostic, socio-economic standard and nationality, that were free of cardio-vascular risk factors) decreasing standard deviation and potentially increasing the power of the study. Another limitation of this study is that it is addressed to young women hospitalized for migraine, and the generalizability to the outpatient MWA female (and male) population is uncertain. Another important limitation of this study is that the non-migraineur dysbiotic population was not studied as a control group, and it is therefore not known whether alterations in gut microbiota could also result in early atherosclerosis in that group. On the other hand, we have previously observed that a gut microbiota imbalance could also be present in various functional pain conditions, such as irritable bowel syndrome, fibromyalgia or chronic pelvic pain. It would be interesting for further research to approach this particular possibility of association between chronic functional pain and early atherosclerosis, via gut microbiota dysbiosis.

However, the most significant limitation of the present pilot study is related to the relatively small sample size of study population, with all potential statistical drawbacks that come from. Future prospective studies with larger participants should address this topic and further validate these preliminary results.

## 5. Conclusions

In this relatively small pilot study of an inpatient population, young female migraineurs with significant alterations of gut microbiota experienced early signs of atherosclerosis and displayed severe migraine disability, as well as multiple biological and clinical particularities.

## Figures and Tables

**Table 1:** Demographic and clinical patients baseline data flow chart.

	DB Negative (*n* = 60)	DB Positive (*n* = 45)	*p*-Value
**Age (years)**	40.33 ± 3.44	39.40 ± 3.48	0.1796
**Urban Location**	81.66%	80%	0.8311
**High Educational level (graduated College)**	71.11%	86.66%	**0.0498**
**Marital situation (married)**	80%	88.88%	0.2238
**Migraine duration (years)**	10.12 ± 3.22	13.91 ± 5.21	**<0.0001**
**MIDAS**	11.25 ± 2.48	16.09 ± 4.78	**<0.0001**
**Body mass index (kg/m^2^)**	24.108 ± 0.696	24.627 ± 1.076	**0.0028**
**Body mass index** **(25–29.9kg/m^2^)**	25%	44.44%	**0.03**
**Systolic blood pressure (mmHg)**	116.25 ± 4.65	118.40 ± 6.15	**0.0438**

Legend: All numeric values are expressed as mean values ± standard deviation, *p* bold means statistically significant. MIDAS = migraine disability assessment score.

**Table 2:** Biological characteristics and CIMT patients data flow chart.

	DB Negative (*n* = 60)	DB Positive (*n* = 45)	*p*-Value
**Hemoglobin (g/dL)**	12.40 ± 1.245	12.27 ± 0.462	0.4102
**Leukocytes/mm^3^**	7.339 × 10^3^ ± 0.676 × 10^3^	7.641 × 10^3^ ± 0.695 × 10^3^	**0.0276**
**Platelets/mm^3^**	235.82 × 10^3^ ± 57.14 × 10^3^	228.00 × 10^3^ ± 44.24 × 10^3^	0.4478
**ALT (IU/dL)**	22.28 ± 3.88	26.13 ± 4.97	**<0.0001**
**Creatinine (mg/dL)**	0.74 ± 0.089	0.76 ± 0.085	0.3743
**Hemoglobin A_1_c (%)**	5.063 ± 0.14	5.328 ± 0.371	**<0.0001**
**Hemoglobin A_1_c:5.7%–6.4%**	5%	20%	**0.0173**
**Ionic calcium (mg/dL)**	8.8817 ± 0.181	9.427 ± 0.568	**<0.0001**
**CRP (mg/dL)**	0.557 ± 0.300	0.687 ± 0.441	0.06
**TNF-alpha (pg/mL^2^)**	6.82 ± 2.57	8.97 ± 2.55	**0.0047**
**CIMT (mm)**	0.767 ± 0.157	0.991 ± 0.25	**<0.0001**
**CIMT>0.9 mm**	15%	40%	**0.039**

Legend: All numerical values are expressed as mean values ± standard deviation; *p* bold means statistically significant, ALT = alanine-aminotransferase, CRP = C reactive protein, TNF-alpha = tumor necrosis factor,-alpha, CIMT = carotid intima media thickness.

**Table 3:** Significant relationships between variables:CIMT and DB in DB+ group.

	Variables	r	*P*
1	CIMT vs. TNF-alpha	0.65	<0.0001
2	CIMT vs. Ca	0.61	<0.0001
3	CIMT vs. HbA_1_c	0.62	<0.0001
4	CIMT vs. DB	0.72	<0.0001
5	CIMT vs. MWA duration	0.35	0.01
6	CIMT vs. MIDAS	0.72	<0.0001
7	CIMT vs. BMI	0.34	0.02
8	DB vs. TNF-alpha	0.62	<0.0001
9	DB vs. BMI	0.43	0.02
10	DB vs. HbA_1_c	0.81	<0.0001
11	DB vs. MIDAS	0.72	<0.0001
12	DB vs.Ca	0.51	0.0003

Legend: CIMT = carotid intima-media thickness, TNF-alpha = tumor necrosis factor-alpha, Ca = calcium, HbA_1_c = glycosylated hemoglobin, DB = dysbiosis, MWA-duration = migraine without aura-duration, MIDAS = migraine disability assessment score, BMI = body mass index.

## References

[1] Kurth T., Chabriat H., Bousser M.G. (2012). Migraine and stroke a complex association with clinical implications. Lancet Neurol..

[2] Sacco S., Ornello R., Ripa P., Pistoia F., Carolei A. (2013). Migraine and hemorrhagic stroke: A meta-analysis. Stroke.

[3] Kurth T., Winter A.C., Dushkes R., Mukamal K.J., Rimm E.B., Willett W.C., Manson J.E., Rexrode K.M. (2016). Migraine and risk of cardio-vascular disease in women: Prospective cohort study. BMJ.

[4] Bigal M.E., Kurth T., Santanello N., Buse D., Golden W., Robbins M., Lipton R.B. (2010). Migraine and cardiovascular disease: A population-based study. Neurology.

[5] Winsvold B.S., Hagen K., Aamodt AHStovner L.J., Holmen J., Zwart J.A. (2011). Headache, migraine and cardiovascular risk factors: The HUNT study. Eur. J. Neurol..

[6] Glass C.K., Witztum J.L. (2001). Atherosclerosis. The road ahead. Cell.

[7] Taylor A.J., Burke A.P., O’Maley P.G., Farb A., Malcom G.T., Smialek J., Virmani R. (2000). A comparison of the Framingham risk index, coronary artery calcification and culprit plaque morphology in sudden cardiac death. Circulation.

[8] Michos E.D., Nasir K., Braunstein J.B., Rumberger J.A., Budoff M.J., Post W.S., Blumenthal R.S.L. (2016). Framingham risk equation underestimates subclinical atherosclerosis risk in asymptomatic women. Atherosclerosis.

[9] Fernandez-Friera R., Fuster V., Lopez-Melgar B., Oliva B., García-Ruiz J.M., Mendiguren J., Bueno H., Pocock S., Ibáñez B., Fernández-Ortiz A. (2017). Normal LDL-cholesterol levels are associated with subclinical atherosclerosis in the absence of risk factors. J. Am. Coll. Cardiol..

[10] Libby P. (2002). Inflammation in atherosclerosis. Nature.

[11] Hansson G.K. (2005). Inflammation, atherosclerosis and coronary artery disease. N. Engl. J. Med..

[12] Campbell L.A., Rosenfeld E. (2015). Infection and ATS development. Arch. Med. Res..

[13] Lindskog A., Bäckhed J.F. (2017). Role of gut microbiota in atherosclerosis. Nat. Rev. Cardiol..

[14] Wang Z., Klipfell E., Bennett B.J., Koeth R., Levison B.S., DuGar B., Feldstein A.E., Britt E.B., Fu X., Chung Y.M. (2011). Gut flora metabolism of phosphatidylcholine promotes cardiovascular disease. Nature.

[15] Lau K., Srivatsav V., Rizwan A., Nashed A., Liu R., Shen R., Akhtar M. (2017). Bridging the gap between gut microbial dysbiosis and cardiovascular diseases. Nutrients.

[16] Ma J., Li H. (2018). The role of gut microbiota in atherosclerosis and hypertension. Front. Pharm..

[17] Li X., Shimizu Y., Kimura I. (2017). Gut microbial metabolite short-chain fatty acids and obesity. Biosci. Microbiota Food Health.

[18] Wang X., Zhang A., Miao J., Sun H., Yan G.L., Wu F.F., Wang X.J. (2018). Gut microbiota as important modulator of metabolism in health and disease. RSC Adv..

[19] Lipton R.B., Bigal M.E., Steiner T.J., Silberstein S.D., Olesen J. (2004). Classification of primary headaches. Neurology.

[20] Stewart W.F., Lipton R.B., Dowson A.J., Sawyer J. (2001). Development and testing of the migraine disability assessment (MIDAS) questionnaire to assess headache related disability. Neurology.

[21] Brown R., Poxton I.R., Wilkinson J.F., Collee J.G., Duguid J.P., Fraser A.G., Marmion B.P. (1999). Centrifuges, colorimeters and bacterial counts. Mackie and McCartney Practical Medical Microbiology.

[22] Sandrin T.R., Goldstein J.E., Shoemaker S. (2013). MALDI TOF MS profiling of bacteria at the strain level: A review. Mass Spectrom. Rev..

[23] Mancia G., Fagard R., Narkiewicz K., Redon J., Zanchetti A., Böhm M., Christiaens T., Cifkova R., De Backer G., Dominiczak A. (2013). 2013 ESH/ESC Guidelines for the management of arterial hypertension. Eur. Heart J..

[24] Dogan S., Duivenvoorden R., Grobbee D., Kastelein J.J., Shear C.L., Evans G.W., Visseren F.L., Bots M.L., Radiance 1 and Radiance 2 Study Groups (2010). Ultrasound protocols to measure carotid intima-media thickness in trials; comparison of reproducibility, rate of progression, and effect of intervention in subjects with familial hypercholesterolemia and subjects with mixed dyslipidemia. Ann. Med..

[25] Goulart A.C., Itamar S., Santo I.S., Bittencourt M.S. (2015). Migraine and subclinical atherosclerosis in the Brazilian Longitudinal Study of Adult Health (ELSA-Brasil). Cephalalgia.

[26] Yilmaz A., Akkucuk M.H., Torun E., Arikan S., Can U., Tekindal M.A. (2019). Migraine and subclinical ATS: Endothelial dysfunction biomarkers and CIMT: A case-control study. Neurol. Sci..

[27] Besir F.H., Koçer A., Dikici S., Yazgan S., Ozdem Ş. (2013). The evaluation of atherosclerosis in migraine patients. Pain Pract..

[28] González-Quintanilla V., Toriello M., Palacio E., González-Gay M.A., Castillo J., Montes S., Martínez-Nieto R., Fernandez J., Rojo A., Gutiérrez S. (2015). Systemic and cerebral endothelial dysfunction in chronic migraine. A case-control study with an active comparator. Cephalalgia.

[29] Saçmacı H., Turan Y. (2019). Increased epicardial fat thickness and carotid intima-media thickness in migraine patients. Neurol. Sci..

[30] Emoto T., Yamashita T., Sasaki N., Hirota Y., Hayashi T., So A., Kasahara K., Yodoi K., Matsumoto T., Mizoguchi T. (2016). Analysis of gut microbiota in coronary artery disease patients: A possible link between gut microbiota and coronary artery disease. J. Atheroscler. Thromb..

[31] Gonzalez A., Hyde E., Sangwan N., Gilbert J.A., Viirre E., Knight R. (2016). Migraines are correlated with higher levels of nitrate-, nitrite-, and nitric oxide-reducing oral microbes in the American gut project cohort. MSystems.

[32] Kamada N., Seo S.U., Chen G.I., Núñez G. (2013). Role of the gut microbiota in immunity and inflammatory disease. Nat. Rev. Immunol..

[33] Parekh P.J., Arusi E., Vinik A.I., Johnson D.A. (2014). The role and influence of gut microbiota in pathogenesis and management of obesity and metabolic syndrome. Front. Endocrinol..

[34] Ajslev T.A., Andersen C.S., Gamborg M., Sørensen T.I., Jess T. (2011). Childhood overweight after establishment of the gut microbiota: The role of delivery mode, pregnancy weight and early administration of antibiotics. Int. J. Obes..

[35] Jess T. (2014). Microbiota, antibiotics and obesity. N. Enl. J. Med..

[36] Falony G., Joossens M., Vieira-Silva S., Wang J., Darzi Y., Faust K., Kurilshikov A., Bonder M.J., Valles-Colomer M., Vandeputte D. (2016). Population-level analysis of gut microbiome variation. Science.

[37] Wang T.K., Bolland M.J., van Pelt N.C., Horne A.M., Mason B.H., Ames R.W., Grey A.B., Ruygrok P.N., Gamble G.D., Reid I.R. (2010). Relationships between vascular calcification, calcium metabolism, bone density and fractures. J. Bone Min. Res..

[38] Silverman N.G., Blaha M.J., Krumholz H.M., Budoff M.J., Blankstein R., Sibley C.T., Agatston A. (2014). Impact of coronary artery calcium on coronary heart disease events in individuals at the extremes of traditional risk factor burden: The multi-ethnic study of atherosclerosis. Eur. Heart J..

[39] Manco M., Putignani L., Bottazzo G.F. (2010). Gut microbiota lipopolysaccharides and innate immunity in the pathogenesis of obesity and cardiovascular risk. Endocr. Rev..

[40] Senthong V., Li X.S., Hudec T., Coughlin J., Wu Y., Levison B., Wang Z., Hazen S.L., Tang W.H. (2016). Plasma TMAO, a gut microbe generated metabolite, is associated with atherosclerotic burden. J. Am. Coll. Cardiol..

[41] Loomba R., Seguritan V., Li W., Long T., Klitgord N., Bhatt A., Dulai P.S., Caussy C., Bettencourt R., Highlander S.K. (2017). Gut microbiom-based metagenomic signature for non invasive detection of advance fibrosis in human nonalcoholic fatty liver disease. Cell Metab..

[42] Georgescu D., Reisz D., Gurban C.V., Georgescu L.A., Ionita I., Ancusa O.E., Lighezan D. (2018). Migraine in young females with irritable bowel syndrome: Still a challenge. Neuropsychiatr. Dis. Treat..

[43] Li X., Watanabe K., Kimura I. (2017). Gut microbiota dysbiosis drives and implies novel therapeutic strategies for diabetes mellitus and related metabolic diseases. Front. Immunol..

[44] Koopen A.M., Groen A.K., Nieuwdorp M. (2016). Human microbiome as therapeutic intervention target to reduce cardiovascular disease risk. Curr. Opin. Lipidol..

[45] Anbazhagan A.N., Priyamvada S., Priyadarshini M. (2017). Gut microbiota in vascular disease: Therapeutic target?. Curr. Vasc. Pharmacol..

[46] Santisteban M.M., Qi Y., Zubcevic J., Kim S., Yang T., Shenoy V., Cole-Jeffrey C.T., Lobaton G.O., Stewart D.C., Rubiano A. (2017). Hypertension-linked pathophysiological alterations in the gut. Circ. Res..

[47] Day R., Stone S., Harper A. (2019). Probiotics for the prophylaxis of migraine: A systematic review of randomized placebo controlled trials. J. Int. Med..

